# Editorial: Investigating Brain Activity After Acquired and Traumatic Brain Injury: Applications of Functional MRI

**DOI:** 10.3389/fneur.2018.00365

**Published:** 2018-05-22

**Authors:** Olga Boukrina, Nancy D. Chiaravalloti, Ekaterina Dobryakova

**Affiliations:** ^1^Stroke, Kessler Foundation, West Orange, NJ, United States; ^2^Neuropsychology and Neuroscience, Kessler Foundation, West Orange, NJ, United States; ^3^Traumatic Brain Injury, Kessler Foundation, West Orange, NJ, United States

**Keywords:** fMRI, traumatic brain injury, stroke, blood oxygen level dependent, editorial, rehabilitation, clinical neuroscience

**Editorial on the Research Topic**

**Investigating Brain Activity After Acquired and Traumatic Brain Injury: Applications of Functional MRI**

Every year, approximately 795,000 people in the United States are affected by stroke and 2.8 million lives are impacted by traumatic brain injury (TBI) (1). Stroke and TBI are also major causes of serious long-term disability, reducing mobility, and impacting thinking, memory, sensation, and emotional functioning. Neuroscience holds great promise in addressing the needs of persons with a history of stroke or TBI by improving the current understanding of brain injury and recovery mechanisms. This is the first step in working to inform and improve the available treatments.

While a great many functional neuroimaging methods exist for studying the healthy brain, such methods have not received widespread acceptance in characterizing patient groups. Several methodological barriers may explain why. First, patient populations can be diverse in terms of injury location and stages of recovery. Accurate measurement and interpretation of functional neuroimaging signal in the damaged brain can also pose a challenge, because stroke and TBI can dramatically alter cerebral blood flow, even in areas that are not affected by a structural lesion (2, 3). Finally, correct interpretation of findings in light of impaired and/or changing behavioral function depends on careful experimental design and precise *a priori* operational definitions of the anticipated effects.

Despite these challenges, or, perhaps, because of them, functional neuroimaging is a promising area of investigation in TBI and stroke. This Research Topic is a collection of original research and review articles focused on functional neuroimaging in persons with TBI and stroke. Below, we highlight a few of the most notable findings and ideas from this collection of articles. Readers are encouraged to access the full text articles for more details.

In one of the two review articles, Medaglia provides an overview of fMRI methodology, analyses, and the caveats of applying these analyses to the injured brain. This includes methods, such as seed-based and voxel-based functional connectivity, effective connectivity, including psychophysiological interactions, causal connectivity, and graph analyses. Medaglia discusses the concept of *functional re-organization*. The term is sometimes used to describe a change in the magnitude of activation or of functional connectivity. It is also used to refer to a re-allocation of function to new brain areas following injury. Medaglia suggests that to improve clarity a precise description of the effect should be provided. Formal tests of re-organization should include a search for areas with activity profile closely resembling that of a damaged area, and with corresponding evidence of recovered behavioral function. Distinguishing different innate recovery mechanisms is especially important in intervention studies, because failing to understand which process may be at work when introducing an intervention, may lead to inadvertent interference with endogenous repair mechanisms.

Nair et al. studied brain activation in acute stroke and healthy older controls participants during a covert verbal fluency task. They controlled for the blood oxygen level dependent (BOLD) response variability across participants using resting state fluctuation amplitude (RSFA) (4). RSFA calibration is thought to eliminate any inter-subject variability due to vascular factors and retain any differences due to neuronal activation factors. They found that after scaling, the BOLD response differences between stroke patients and healthy controls were eliminated. This finding suggests that some of the group differences were due to vascular variables. Additionally, some fine-tuning may be required when scaling with RSFA, perhaps scaling by brain region, rather than across the whole brain.

Bernier et al. applied graph theory to a data set of healthy and TBI subjects with moderate/severe TBI. Their aim was to determine if loss of network differentiation accounts for changes in brain connectivity, specifically hyperconnectivity. This hypothesis was examined within the default mode (DMN) and the task positive network. Supporting other results in the field, they observed hyperconnectivity within the DMN and task positive networks. DMN hyperconnectivity was found to be associated with higher scores on the standardized working memory measure. Thus, the work of these authors demonstrates how fMRI and connectivity analyses can inform the cognitive profile observed following TBI.

The second review in the Research Topics explores a common deficit in TBI. Namely, cognitive control, an executive function that is generally necessary for switching between habitual and goal-directed behavior. In his review, Scheibel talks about functional neuroimaging studies of cognitive control in mild TBI (mTBI). The review draws attention to how the fMRI findings are mixed, with reports of decreased as well as increased brain activation in mTBI, and urges for future studies to aim at recruiting more homogenous samples, as the mixed findings might be explained by the presence of comorbidies in TBI samples.

The original research article by Saleh et al. explored how different approaches to rehabilitation of hand function after stroke can alter brain activity across the sensorimotor brain networks and demonstrates network re-organization discussed in the Medaglia review. Both treatment approaches tested in the study improved hand function. However, only the robot-assisted virtual reality group showed reduction of activity and re-lateralization of activation to ipsilesional cortex, a pattern associated with better arm function in this study and with positive recovery in other studies (5).

A contribution by Möller et al. used arterial spin labeling (ASL) fMRI to examine fatigue in mTBI during psychomotor vigilance task performance. The mTBI participants showed different patterns of brain activation compared to healthy controls, in addition to higher self-reported fatigue and reductions in performance as the task progressed (fatigability). Together with the self-reported fatigue ratings and task performance, the ASL results suggested the engagement of disparate functional networks compared in mTBI.

fMRI research in stroke and TBI poses a unique set of challenges to researchers. The articles assembled in this Research Topic address some of these challenges. Using methods designed to work in patients with brain lesions, using appropriate controls, and applying network neuroscience tools are a few of the promising solutions. This topic is an important frontier in neuroscience research today offering tangible benefits for public health and is a potential area of growth in the coming years.

## Author Contributions

OB, ED, and NC participated in manuscript writing and editing.

## Conflict of Interest Statement

The authors declare that the research was conducted in the absence of any commercial or financial relationships that could be construed as a potential conflict of interest.
